# Supplementary material for: Hypersensitivity of the vimentin cytoskeleton to net-charge states and Coulomb repulsion
Source: bioRxiv. 2024 Jul 11:2024.07.08.602555. Preprint. [Version 1] doi: 10.1101/2024.07.08.602555 (PMC11257561; doi:10.1101/2024.07.08.602555)
Supplement: 1 [file NIHPP2024.07.08.602555V1-supplement-1.pdf]

bioRxiv preprint doi: https://doi.org/10.1101/2024.07.08.602555; this version posted July 11, 2024. The copyright holder for this preprint (which was not certified by peer review) is the author/funder, who has granted bioRxiv a license to display the preprint in perpetuity. It is made available under aCC-BY 4.0 International license.

Supporting Information

Material and methods

Cell culturing. COS-7 and PtK2 cells (University of California Berkeley Cell Culture Facility) were cultured in Dulbecco's Modified Eagle's Medium (DMEM, high glucose) with the addition of 10% fetal bovine serum (FBS), 1× non-essential amino acids (NEAA), and 1× GlutaMAX Supplement (for PtK2 cells only), in 5% CO_2 at 37 °C. For live-cell and SM*d*M imaging, cells were plated in 8-well chambered coverglasses (Nunc Lab-Tek II). For STORM imaging, cells were plated on 18 mm #1.5 glass coverslips. Primary rat hippocampus astrocytes were from BrainBits LLC, and were cultured in the NbAstro medium (BrainBits) and plated on coverslips coated with poly-D-lysine, per recommended protocols.

Plasmid constructs and transfection. Vimentin-mEos3.2 (Addgene #57485), vimentin-mCherry (Addgene #55156), and keratin-mEmerald (KRT18-mEmerald; Addgene #54134) plasmids were gifts from Michael Davidson. Vimentin-(-5)-mEos3.2 and vimentin-(+6)-mEos3.2 (**Table S1**) were prepared by first cutting out the mEos3.2 from Vimentin-mEos3.2 with BamHI and NotI restriction enzymes. The plasmids were then ligated through Gibson Assembly with the PCR product of vimentin-mEos3.2 and the desired linkers. The constructed plasmids were amplified in XL1-Blue cells and extracted with the QIAprep Spin Miniprep Kit (QIAGEN). The DNA sequences were verified by Sanger sequencing at the UC Berkeley DNA Sequencing Facility. Cells were transfected with the Neon Transfection System (ThermoFisher) or Lipofectamine 3000 (ThermoFisher) according to the recommended protocols, 48-72 hours before imaging.

Live-cell imaging. Unless otherwise mentioned, for live-cell imaging, the cell culture medium was first replaced with an isotonic imaging buffer (*14*), Leibovitz's L-15 medium (Gibco 21083027) supplemented with 20 mM HEPES (pH 7.3; Gibco 15630106). Dulbecco's phosphate-buffered saline (DPBS) was from Gibco (14040133). The 5 mM pH = 7.3 phosphate buffer was prepared by diluting a 0.1 M phosphate buffer (Sigma-Aldrich, P5244). The 5 mM pH = 5.0 acetate buffer was prepared by diluting a 0.1 M potassium acetate buffer (Sigma-Aldrich, SAE0157). The pH = 9.0 and 10.0 CAPSO buffers were prepared by mixing the CAPSO acid (Sigma-Aldrich C2278) and sodium salt (Sigma-Aldrich C2154) at different ratios and diluting to 5 mM. Triton X-100, saponin, glucose, sorbitol, and KCl (Sigma-Aldrich) were added to the described amounts. Epifluorescence imaging was performed on a Nikon Ti-E inverted fluorescence microscope with an oil-immersion objective (Nikon CFI Plan Apochromat λ 100×, NA 1.45) or an Olympus IX73 inverted epifluorescence microscope with a water-immersion objective (Olympus, UPLSAPO60XW, NA 1.2).

SM*d*M of live cells. SM*d*M was performed on a setup based on a Nikon Ti-E inverted fluorescence microscope, as described previously (*14, 15*). Briefly, excitation and photoactivation lasers at 561 and 405 nm were focused at the back focal plane of an oil-immersion objective lens (Nikon CFI Plan Apochromat λ 100×, NA 1.45) toward the edge of the objective lens to illuminate a few micrometers into the cell. Single-molecule images were continuously

bioRxiv preprint doi: https://doi.org/10.1101/2024.07.08.602555; this version posted July 11, 2024. The copyright holder for this preprint (which was not certified by peer review) is the author/funder, who has granted bioRxiv a license to display the preprint in perpetuity. It is made available under aCC-BY 4.0 International license.

recorded in the wide field with an EM-CCD (iXon Ultra 897, Andor) at a framerate of 110 Hz. The 561 nm excitation laser was repeatedly applied as tandem excitation pulses of $\tau = 500$ µs duration across paired camera frames with a center-to-center separation of $\Delta t = 1$ ms, so that single-molecule displacements were recorded across the paired frames for the fixed 1 ms time window. For analysis, the single-molecule displacements accumulated over a given period were first spatially binned onto a 240×240 nm^2 grid. For each spatial bin, the accumulated single-molecule displacements were fitted through maximum likelihood estimation (MLE) to a modified two-dimensional random walk model with the probability distribution $P(r) = \frac{2r}{a}\exp(-\frac{r^2}{a}) + br$, where r is the single-molecule displacement, $a = 4D\Delta t$, D being the diffusion coefficient, and b accounts for the background. By thus determining the D values for each spatial bin, color-coded SM*d*M maps of local D were generated.

Immunofluorescence and 3D-STORM of fixed cells. Three-dimensional stochastic optical reconstruction microscopy (3D-STORM) was performed for fixed cells as described previously (*11, 12, 54*). Briefly, cells were fixed with 3% (w/v) paraformaldehyde and 0.1% (w/v) glutaraldehyde in DPBS for 20 min. After reduction with a freshly prepared 0.1% sodium borohydride solution in PBS for 5 min, the sample was permeabilized and blocked in a blocking buffer (3% w/v bovine serum albumin (BSA) and 0.1% v/v Triton X-100 in DPBS) for 1 h. Afterward, the cells were incubated with primary antibodies in the blocking buffer for 12 h at 4 °C. After washing in a washing buffer (0.3% w/v BSA and 0.01% v/v Triton X-100 in DPBS) three times, the cells were incubated with dye-labeled secondary antibodies for 1 h at room temperature. Then, the samples were washed three times with the washing buffer and three times with PBS. Primary antibodies used: rabbit anti-vimentin (Cell Signaling Technology, 5741, 1:100), rabbit anti-GFAP (Proteintech, 16825-1-AP, 1:200), and mouse anti-pan cytokeratin (Sigma-Aldrich, C2562, 1:100). Secondary antibodies used: Alexa Fluor 647-labeled goat anti-rabbit (Invitrogen, A21245) for STORM imaging and donkey anti-mouse (Jackson ImmunoResearch, 715-005-151) conjugated with CF568 succinimidyl ester (Biotium, 92131) for epifluorescence imaging in a second color channel when needed. The immunolabeled sample was imaged in a Tris-Cl buffer (pH 7.5) containing 100 mM cysteamine, 5% glucose, 0.8 mg/mL glucose oxidase, and 40 µg/mL catalase. The 647 nm laser illuminated the sample at ~2 kW/cm^2, which photoswitched most of the labeled dye molecules into a dark state while allowing a small, random fraction of molecules to emit across the wide field over different camera frames. Single-molecule emission was passed through a cylindrical lens of focal length 1 m to introduce astigmatism, and recorded with an Andor iXon Ultra 897 EM-CCD camera at a framerate of 110 Hz. A total of ~50,000 frames were recorded for each STORM run. The recorded single-molecule images were localized and rendered as super-resolution images as described previously (*11, 12*).

bioRxiv preprint doi: https://doi.org/10.1101/2024.07.08.602555; this version posted July 11, 2024. The copyright holder for this preprint (which was not certified by peer review) is the author/funder, who has granted bioRxiv a license to display the preprint in perpetuity. It is made available under aCC-BY 4.0 International license.

Table S1. List of protein amino acid (AA) sequences and estimated net charges at pH = 7.3 per Protein Calculator v3.4 (http://protcalc.sourceforge.net).

Protein	Protein sequence	Size (AA)	Net charge
**Vimentin**	STRSVSSSSYRRMFGGPGTASRPSSSRSYVTTSTRTYSLGSALRPSTSRSLYASS<br>PGGVYATRSSAVRLRSSVPGVRLLQDSVDFSLADAINTEFKNTRTNEKVELQELN<br>DRFANYIDKVRFLEQQNKILLAELEQLKGQGKSRLGDLYEEEMRELRRQVDQLTN<br>DKARVEVERDNLAEDIMRLREKLQEEMLQREEAENTLQSFRQDVDNASLARLDLE<br>RKVESLQEEIAFLKKLHEEEIQELQAQIQEQHVQIDVDVSKPDLTAALRDVRQQY<br>ESVAAKNLQEAEEWYKSKFADLSEAANRNNDALRQAKQESTEYRRQVQSLTCEVD<br>ALKGTNESLERQMREMEENFAVEAANYQDTIGRLQDEIQNMKEEMARHLREYQDL<br>LNVKMALDIEIATYRKLLEGEESRISLPLPNFSSLNLRETNLDSLPLVDTHSKRT<br>LLIKTVETRDGQVINETSQHHDDLE	465	−18.4
**mEos3.2**	MSAIKPDMKIKLRMEGNVNGHHFVIDGDGTGKPFEGKQSMDLEVKEGGPLPFAFD<br>ILTTAFHYGNRVFAKYPDNIQDYFKQSFPKGYSWERSLTFEDGGICNARNDITME<br>GDTFYNKVRFYGTNFPANGPVMQKKTLKWEPSTEKMYVRDGVLTGDIEMALLLEG<br>NAHYRCDFRTTYKAKEKGVKLPGAHFVDHCIEILSHDKDYNKVKLYEHAVAHSGL<br>PDNARR	226	+0.9
**Vimentin-mEos3.2**	**Vimentin**-GDPPVAT-**mEos3.2**	698	−18.3
**Vimentin-(−5)-mEos3.2**	**Vimentin**-GDPPEADAEAE-**mEos3.2**	702	−22.3
**Vimentin-(+6)-mEos3.2**	**Vimentin**-GKKGRARKRAG-**mEos3.2**	702	−11.3
**KRT18 (example of Type I keratin)**	SFTTRSTFSTNYRSLGSVQAPSYGARPVSSAASVYAGAGGSGSRISVSRSTSFRG<br>GMGSGGLATGIAGGLAGMGGIQNEKETMQSLNDRLASYLDRVRSLETENRRLESK<br>IREHLEKKGPQVRDWSHYFKIIEDLRAQIFANTVDNARIVLQIDNARLAADDFRV<br>KYETELAMRQSVENDIHGLRKVIDDTNITRLQLETEIEALKEELLFMKKNHEEEV<br>KGLQAQIASSGLTVEVDAPKSQDLAKIMADIRAQYDELARKNREELDKYWSQQIE<br>ESTTVVTTQSAEVGAAETTLTELRRTVQSLEIDLDSMRNLKASLENSLREVEARY<br>ALQMEQLNGILLHLESELAQTRAEGQRQAQEYEALLNIKVKLEAEIATYRRLLED<br>GEDFNLGDALDSSNSMQTIQKTTTRRIVDGKVVSETNDTKVLRH	429	−10.3
**KRT1 (example of Type II keratin)**	SRQFSSRSGYRSGGGFSSGSAGIINYQRRTTSSSTRRSGGGGGRFSSCGGGGGSF<br>GAGGGFGSRSLVNLGGSKSISISVARGGGRGSGFGGGYGGGGFGGGGFGGGGFGG<br>GGIGGGGFGGFGSGGGGFGGGGFGGGGYGGGYGPVCPPGGIQEVTINQSLLQPLN<br>VEIDPEIQKVKSREREQIKSLNNQFASFIDKVRFLEQQNQVLQTKWELLQQVDTS<br>TRTHNLEPYFESFINNLRRRVDQLKSDQSRLDSELKNMQDMVEDYRNKYEDEINK<br>RTNAENEFVTIKKDVDGAYMTKVDLQAKLDNLQQEIDFLTALYQAELSQMQTQIS<br>ETNVILSMDNNRSLDLDSIIAEVKAQYEDIAQKSKAEAESLYQSKYEELQITAGR<br>HGDSVRNSKIEISELNRVIQRLRSEIDNVKKQISNLQQSISDAEQRGENALKDAK<br>NKLNDLEDALQQAKEDLARLLRDYQELMNTKLALDLEIATYRTLLEGEESRMSGE<br>CAPNVSVSVSTSHTTISGGGSRGGGGGGYGSGGSSYGSGGGSYGSGGGGGGGRGS<br>YGSGGSSYGSGGGSYGSGGGGGGHGSYGSGSSSGGYRGGSGGGGGGSSGGRGSGG<br>GSSGGSIGGRGSSSGGVKSSGGSSSVKFVSTTYSGVTR	643	+2.2
**GFAP**	MERRRITSAARRSYVSSGEMMVGGLAPGRRLGPGTRLSLARMPPPLPTRVDFSLA<br>GALNAGFKETRASERAEMMELNDRFASYIEKVRFLEQQNKALAAELNQLRAKEPT<br>KLADVYQAELRELRLRLDQLTANSARLEVERDNLAQDLATVRQKLQDETNLRLEA<br>ENNLAAYRQEADEATLARLDLERKIESLEEEIRFLRKIHEEEVRELQEQLARQQV<br>HVELDVAKPDLTAALKEIRTQYEAMASSNMHEAEEWYRSKFADLTDAAARNAELL<br>RQAKHEANDYRRQLQSLTCDLESLRGTNESLERQMREQEERHVREAASYQEALAR<br>LEEEGQSLKDEMARHLQEYQDLLNVKLALDIEIATYRKLLEGEENRITIPVQTFS<br>NLQIRETSLDTKSVSEGHLKRNIVVKTVEMRDGEVIKESKQEHKDVM	432	−12.1

bioRxiv preprint doi: https://doi.org/10.1101/2024.07.08.602555; this version posted July 11, 2024. The copyright holder for this preprint (which was not certified by peer review) is the author/funder, who has granted bioRxiv a license to display the preprint in perpetuity. It is made available under aCC-BY 4.0 International license.

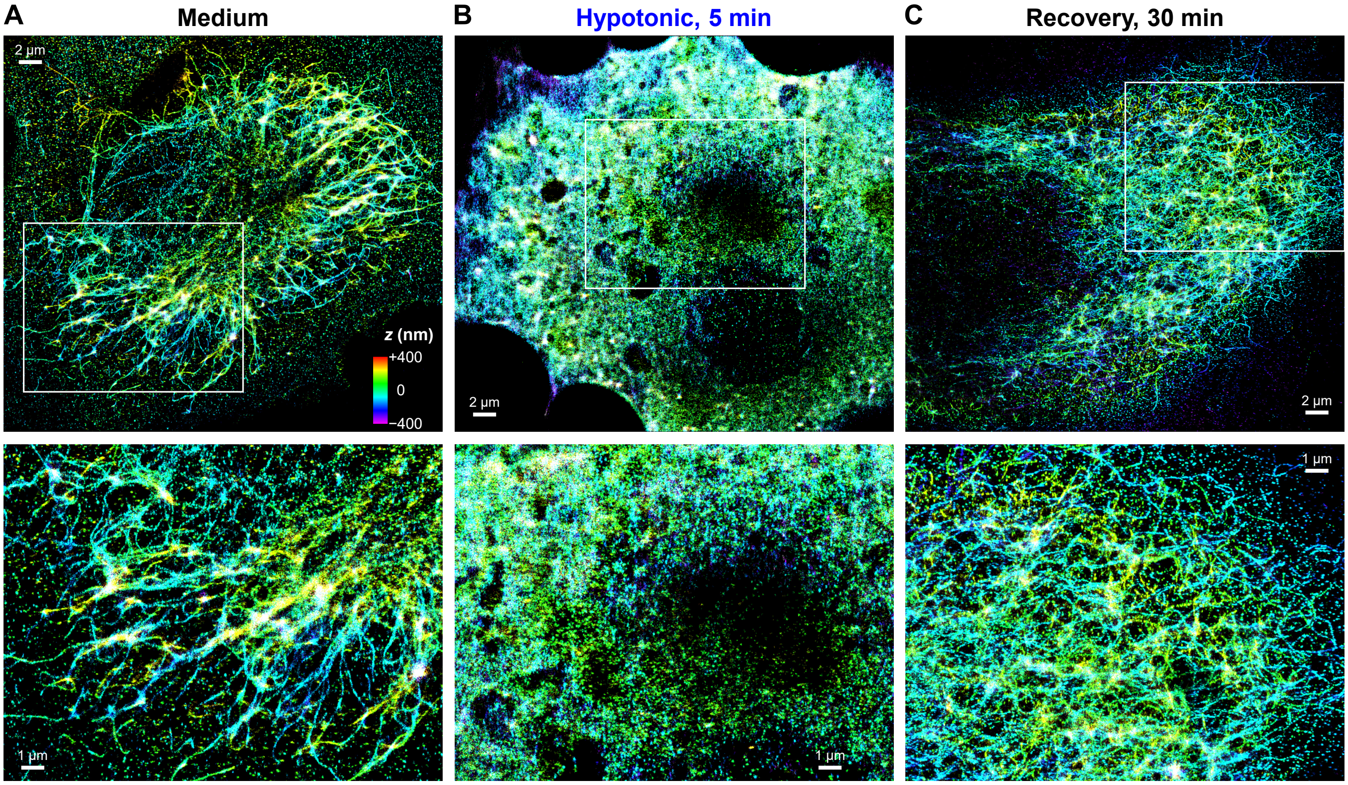

Figure S1. Representative 3D-STORM images of immunolabeled vimentin in COS-7 cells. (**A**) An untreated cell. The bottom figure is a zoom-in of the boxed region in the top figure. (**B**) Another cell, fixed after hypotonic treatment with water for 5 min. The zoom-in image shows the complete disassembly of filaments. (**C**) Another cell, after hypotonic treatment with water for 5 min, but then allowed to recover for 30 min in the regular cell culture medium in the incubator, before being fixed and labeled. Color encodes axial (depth) position, according to the color scale shown in (A).

bioRxiv preprint doi: https://doi.org/10.1101/2024.07.08.602555; this version posted July 11, 2024. The copyright holder for this preprint (which was not certified by peer review) is the author/funder, who has granted bioRxiv a license to display the preprint in perpetuity. It is made available under aCC-BY 4.0 International license.

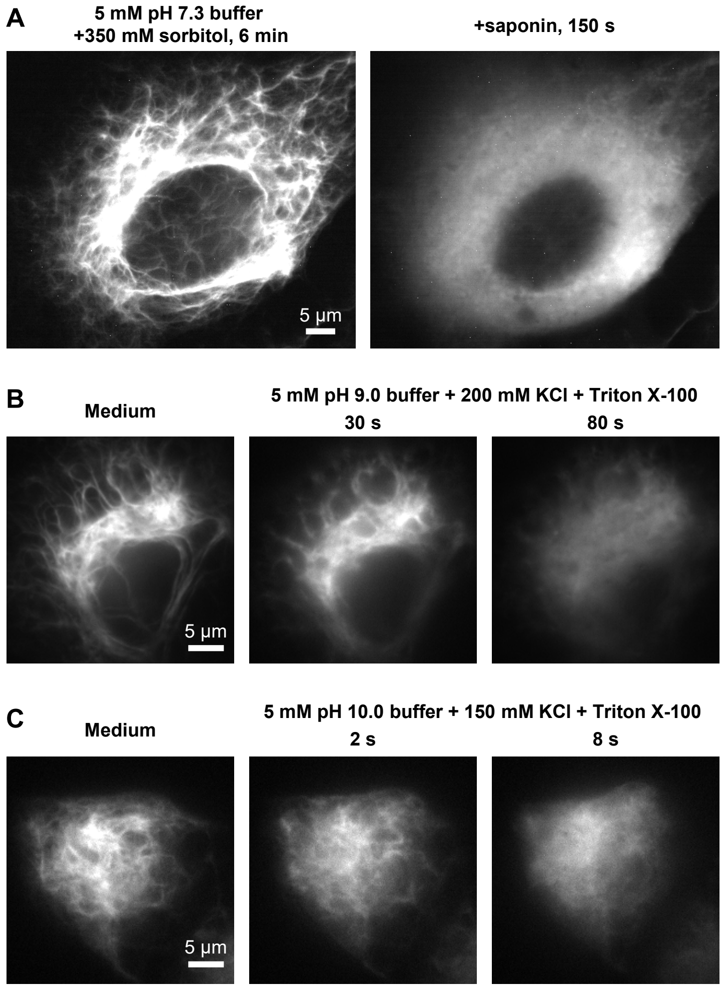

Figure S2. Additional fluorescence micrographs of vimentin-mEos3.2 in COS-7 cells after permeabilization under different ionic strengths and pHs. (**A**) Fluorescence micrographs of a cell (**i**) 6 min in a 5 mM phosphate buffer (pH = 7.3) with 350 mM sorbitol added and (**ii**) 150 s after next adding 50 µg/mL saponin into the medium. (**B**) Fluorescence micrographs of another cell before and after replacing the cell medium with a 5 mM CAPSO buffer (pH = 9.0) with the addition of 200 mM KCl and 0.2% Triton X-100, at 30 s and 80 s. (**C**) Fluorescence micrographs for another cell before and after replacing the cell medium with a 5 mM CAPSO buffer (pH = 10.0) with the addition of 150 mM KCl and 0.2% Triton X-100, at 2 s and 8 s.

Movie S1. Consecutive time series of the two-color live-cell data in **Fig. 4B**, shown as overlaid and separate color channels for vimentin-mCherry (magenta) and keratin-mEmerald (green). Time 0 corresponds to when the medium is changed to the 5 mM phosphate buffer. Scale bar: 5 µm.
